# Validation of the factor structure and psychometric characteristics of the Arabic adaptation of the sense of coherence SOC-13 scale: a confirmatory factor analysis

**DOI:** 10.1186/s40359-022-00826-4

**Published:** 2022-05-03

**Authors:** Fatimah Sayer Alharbi, Abdulaziz I. Aljemaiah, Mugtaba Osman

**Affiliations:** 1 Department of Psychology, College of Education, Nourah’s Health Program, Princess Nourah Bint Abdulrahman University, PO Box 84428, Riyadh, 11671 Saudi Arabia; 2 Armed Forces Centre for Psychiatric Care, Prince Mansour Military Hospital, Al-Matar Street, Al-Faiysaliyah District, Taif, Saudi Arabia

**Keywords:** Sense of coherence, SOC-13 scale, Arabic SOC-13 scale, Confirmatory factor analysis, Reliability, Psychometric analysis, Saudi Arabia

## Abstract

**Background:**

The sense of coherence is as focused on one’s awareness of the level of pervasive, enduring, and dynamic feelings. Stronger sense of coherence leads to better physical and mental health and promotes recovery from life stressors. Sense of coherence-13 (SOC-13) is a 13-item valid and reliable measure for individual’s healthy living. However, the factor structure of SOC-13 was criticized in several cultures and languages. The current study was set to explore the factor structure of an Arabic adaptation for SOC-13.

**Methods:**

This cross-sectional study of the SOC-13 included (n = 1235) Arabic speaking individuals. We used confirmatory factor analysis to contrast unidimensional, bidimensional, three-dimensional, and four-dimensional factor structure for the SOC-13. We carried out measurement invariance analysis across age and gender groups to examine the stability of fit indices among participants’ subgroups.

**Results:**

We found the reliability coefficient to be 0.82, indicative of good internal consistency. The three-factor structure, after modification of items 1, 2, and 3 was the best-fitting factor model. However, measurement invariance was indicative of discrepancy for the three-factor model between genders and age classes. The mean overall SOC-13 total score in our sample was 52.1 (SD = 16.1).

**Conclusions:**

The SOC-13 showed acceptable psychometric properties in terms of internal consistency and a modified three-factor structure in its Arabic version. However, the reliability of the three underlying dimensions was sub-optimum. Moreover, the three-factor structure requires modification by either removing the first three problematic items or allowing the residuals to correlate.

**Supplementary Information:**

The online version contains supplementary material available at 10.1186/s40359-022-00826-4.

## Background

Sense of coherence SOC was initially described by Aaron Antonovsky in the late 1970s [1], to evolve substantially afterwards over the subsequent 4 decades. The sense of coherence was viewed first as an “individual property” [1]. However, a decade later, the concept was widened to include the nuclear family of the individual [2]. It was further expanded by [2] to focus on one’s awareness of the level of pervasive, enduring, and dynamic feelings.

Furthermore, the twenty-first century research extended sense of coherence to cover wider organizations in which the individual and family would interact, affect, and affect by, for instance their workplace organization [3]. Stronger sense of coherence meant better physical and mental health and more use of mature defence mechanisms [4]. Strong sense of coherence has a role in mediating recovery from substantial stressors such as poly-victimization [5].

The principal theoretical framework that underpins the measurement power of SOC scale is the so-called Generalized Resistance Resource (GRR). GRR is a term proposed by *Antonovsky* [1979] [1], well prior to the development of the Sense of Coherence model. GRR constitutes the adaptive coping abilities of the individual and society and, hence, it potentiates the development of a sense of coherence. GRRs facilitate person’s ability to rebound. The twelve GRR factors, upon which the SOC-29 and the SOC-13 were designed comprise a range of cultural, societal, philosophical and biopsychological resources individuals could use [6].

Based on his broad definition of the sense of coherence, *Antonovsky* [1987] [2] proposed a 29-item scale for its measurement. He called it the Orientation to Life Questionnaire with the subscale of comprehensibility, the cognitive dimension, to be measured collectively by 11 items. Comprehensibility was defined by *Eriksson* [2017] [7] as “the extent to which one perceives internal and external stimuli as rationally understandable, and as information that is orderly, coherent, clear, structured rather than noise—that is, chaotic, disordered, random, unexpected, and unexplained”. The context of oneself and the role one plays in that context would be far more understandable should one have mastered the faculty of building a structure out of chaos. Clearly, the ability to manage stresses is substantially improved once one makes sense of them. Therefore, comprehensibility was regarded by researchers as a prerequisite for adaptive coping [7].

The subdimension of manageability, the behavioural dimension, must be measured collectively by 10 items. Manageability can be defined as “the degree to which one feels that there are resources at one’s disposal that can be used to meet the requirements of the stimuli one is facing” [7]. Such resources are divided into two categories: formal (such as public services and charitable entities) and informal (family and friends, for instance).

The remaining 8 items were set to measure the meaningfulness sub-score, which constitutes the motivational dimension. Meaningfulness is composed of motivation, commitment, dedication, will for energy spending, and the emotional meaning of being able to solve every-day problems. Meaningfulness views daily problems as “challenges” rather than “burdens” [7].

SOC-13 is a 13-item self-report measure developed by [Antonovsky., 1993] [8] from the longer SOC-29 scale to briefly assess the extent of emotional control. The SOC-13, given its strong psychometric properties, was used extensively in several settings [9]. All items are answered on a 7-point Likert scale over a semantic differential scale with two extreme anchoring phrases. Five items (namely, items 1, 2, 3, 7, and 10) are reversed in scoring. The total score would, hence, range between 13 and 91. Low scores indicate low/weak sense of coherence, and high scores mean high/strong sense of coherence. There are 5 comprehensibility, 4 manageability, and 4 meaningfulness items, however, the scale gives a single score of sense of coherence. Example items for *comprehensibility* include, “Do you have the feeling that you are in an unfamiliar situation and don’t know what to do?’’ and “Does it happen that you have feelings inside you would rather not feel?”. Items identifying *manageability* include, “Do you have the feeling that you are being treated unfairly?” and “Has it happened that people whom you counted on disappointed you?”. *Meaningful* items include, “Do you have the feeling that you don’t really care about what goes on around you?” and “How often do you have the feeling that there’s little meaning in the things you do in your daily life?”. To clarify further, the comprehensibility (consists of items: 2, 6, 8, 9, and 11), manageability (consists of items: 3, 5, 10, and 13), and meaningfulness (consists of items: 1, 4, 7, and 12).

The concept of sense of coherence was examined in several contexts. A recent study, for instance, examined the association between the sense of coherence and maternal attachment to their babies [10]. Sense of coherence was associated positively with both maternal attachment to the newly born offspring and the acceptance of maternal role.

Many studies attempted to examine the psychometric properties of the SOC-13 in its various formats. The SOC-13 was first proposed by *Antonovsky* [1987] [2]. The SOC-13 questionnaire was tested and validated in several languages. The factor structure was shown to be consistent [11]. Recently, an orphan study [12] attempted to explore the validity of an Arabic version of SOC-13 among children in the UAE. They found the Cronbach’s alpha to be good at 0.75. No attempt was made to examine the factor structure in depth. This is what we intend to do in the current investigation. In-depth factor structure analysis in adults living in Saudi Arabia.

Only a dearth of studies explored the psychometric properties of the SOC-13 (or its SOC-29 parent) or even used it in the Arabic language. On the other hand, several global studies examined multicultural adaptations of the SOC-13. The remaining question is whether the thirteen items that constitute the SOC-13 scale should be regarded as a collective single dimension as envisioned by the originator of the scale [*Antonovsky* 1987] [2] and suggested by the findings of a chain of studies [8], or to approve of the three-dimensional structure supported by many other studies on the same SOC-13 scale [13].

*Al-Yateem *et al*.* [2020] [12] realized how the wellbeing of Arabic-speaking adolescents would be improved substantially if their sense of coherence was supported to grow. In their study, they set to evaluate the reliability and validity estimates of the Arabic adaptation of the SOC scale. They also wanted to contribute normative values for the SOC scale in the Arabic speaking United Arabic Emirates UAE population. Encouragingly, they established a very good internal consistency estimate for the SOC questionnaire, with a Cronbach's alpha of 0.75. They set the normative SOC average at 57.4, pioneering the SOC research among Arabic communities.

*Lerdal *et al*.* [2017] [14] surveyed (n = 428) irritable bowel syndrome patients using the Norwegian version of the SOC-13. They adopted a Rasch analysis to examine a range of psychometric properties that included internal validity, functioning, differential item functioning, person-response validity, and person-separation reliability. They found that the SOC-13 functions with good and related to the collapsing categories at the low end of the seven-item Likert style of the scale. They found that two items did not constitute a good fit in the total SOC-13, namely, item 1: “Do you have the feeling that you don’t really care about what goes on around you” and item 5: “Do you have the feeling that you are being treated unfairly?”. Removal of both substantially improved the overall fit. SOC-13, in their sample, did not satisfy characteristics of person-response validity or one-dimensionality.

A recent Slovenian study [15] attempted to evaluate the psychometric properties of SOC-13. The investigation included (n = 134) Multiple Sclerosis patients. With a Cronbach’s alpha of 0.83, they found the SOC-13 to be of good overall internal consistency. However, the reliability was not so good for the three subscores, therefore the authors encouraged the use of the overall summary score for the SOC-13 rather than summary statistics for the less reliable subscores. Manageability dimension had a Cronbach’s alpha of 0.66, improved to 0.69 for the meaningfulness dimension, and to 0.79 for the comprehensibility dimension. The three-factor structure provided an acceptable fit to the data, as the RMSEA was 0.059. The three factors intercorrelated substantially. However, the confirmation of three-factor structure required the authors to correlate the residuals of two-item pairs. They allowed the residuals of (Item 2: “Has it happened in the past that you were surprised by the behaviour of people whom you thought you knew well?” and Item3: “Has it happened that people whom you counted on disappointed you?”) to correlate, as they addressed participants’ expectations regarding the people around them. They also allowed residual correlations of (item 4: “Until now your life has had clear goals and purposes” and item 13: “How often do you have feelings that you’re not sure you can keep under control?”) as they were both concerned with the management of life situations in terms of clarity of goals and control of feelings.

A group of researchers from India’s Mangalore’s University [16] explored the psychometric characteristics of SOC-13 among second-year degree students. They focused their assessment on its comprehensiveness, appropriateness, understandability, and relevance. The Cronbach's alpha estimate of the internal consistency was 0.76. Estimate for split-half reliability was 0.71 and the estimate for Guttman split-half reliability was 0.70. SOC-13 was found to have good Test–retest reliability (0.71, *P* < 0.01). They found that 41% of the SOC-13 variability can be explained by the three-factor solution.

In Australia, a study examined the psychometric properties in a group of (n = 718) pregnant women [17]. That was based on the established positive association between sense of coherence and childbearing health [18]. They estimated the mean SOC-13 score at 67.5 (SD = 10.9). They were faced with difficulty in establishing the construct validity of the SOC 13 was difficult to establish. Construct validity was defined during their investigation to mean “the ability of an instrument to measure an abstract concept”. They decided to remove four items before they were content with the construct validity of the SOC-13 scale, namely, items 2, 3, 7, and 9. They noted sound criterion validity for the SOC-13 before and after removal of the four problematic items, as well as internal reliability.

One of the earliest attempts at using Arabic SOC scale among Arabic speaking communities was the study conducted by Cohen and Savaya [2003] [19]. They survey 306 divorced Muslim Arabs in Israel. Their results indicated that the sense of coherence is closely linked to mental health, however, they remain independent constructs. These were certainly important findings. However, the authors did not report on the psychometric properties of the SOC-29 scale they used. They focused the analysis primarily on the correlation between sense of coherence and mental health among the divorced Arab community who participated in the study.

Very recently, *Abu-Kaf and Khalaf* [2020] [20] evaluated the relationship between depression and acculturative stress in (n = 170) Arab undergraduate students. They used the SOC-13 scale to assess the protective impact of the sense of coherence on the depression-acculturative stress association. They viewed the sense of coherence as a healthy way of seeing life and coping with its stresses [8]. Arab students in higher academic years demonstrated significantly better sense of coherence and less depressive symptoms. Sense of coherence was negatively associated with avoidant coping and depressive symptoms but was positively associated with active coping styles. They demonstrated that the sense of coherence constituted an indirect link between depressive symptoms and active coping (in male students) and avoidant coping (among female students).

A sample of (n = 566) dentistry students in Istanbul University completes the SOC-13 scale [21]. The median score was found to be 56, with scores ranging between 22 and 91. Clearly, a strong sense of coherence was associated positively with better oral health-related behaviours and lower levels of stress.

A Farsi adaptation of the SOC-13 was validated favourably among a sample of (n = 375) Iranian undergraduate students [22]. The estimate for the internal consistency of the Farsi SOC-13 was good (Cronbach’s alpha = 0.77). The correlation between test-retests was statistically significant (r = 0.66). Factor analysis extracted four factors, which explained 53.49% of the total variability.

The main objective of our current investigation is the comprehensive psychometric evaluation of the Arabic version of SOC-13 in terms of underlying three-factor structure, internal consistency, and reliability. We also aimed at evaluating the effect of background demographic factors on the SOC-13 in a large-scale sample for the Saudi public.

## Methods

### Eligibility criteria and data collection procedure

Demographic and SOC-13 data were collected through a predesigned questionnaire mounted onto an online portal that was distributed among potential respondents by sharing the unique link using mass email and social networking media.

The participants were form the general public of citizens and residents in Saudi Arabia. Exclusion criteria were entering incomplete or inappropriate responses to the SOC-13 questionnaire. We also excluded participants under the age of 18.

### Measures

We used the Arabic version SOC-13, which was obtained from the original authors’ website with permission. The Arabic version was obtained whole from STARS: Society for Theory And Research on Salutogenesis website (https://www.stars-society.org/kccmhshvugiq). We were not directly involved in the translation or adaptation of the original SOC-13 to Arabic.

### Data analyses

The dataset in total was automatically transferred into Microsoft Excel system sheet. The advanced statistical methods to examine the psychometric properties included confirmatory factor analysis (CFA) using R-Statistical software. CFA is a statistical technique that examines the association between latent variables and relates that with a pre-set theoretic covariance structure [23] and is an established statistical method to evaluate construct validity [24]. We examined different factor structures including one-factor, two-factor, three-factor, and four-factor structures. To examine the potential baseline differences in terms of model-fit, we ran a measurement invariance anlysis across the different age, marital status, gender, employment, and psychiatric history groups. We compared the models in terms of absolute fit indices that include the Chi-Squared test, root mean square error of approximation, RMSEA, comparative fit index, CFI, goodness-of-fit index, GFI, root mean square residual RMR, and standardized root mean square residual SRMR.

For a good model-fit a chi-squared value needs to be non-significant statistically. The cut-off for comparative fit index (CFI) was set at > 0.95, and for Tucker-Lewis index (TLI) t > 0.95, and root mean square error of approximation (RMSEA) required to be < 0.06 [25]. The cut-off for acceptable model-fit is when RMSEA is < 0.08 [26].

Normality was tested by visualizing the data graphically using histogram and applying shipro wilk test (for the SOC-13 the test *P* value was 0.1063 indicative of satisfactory normality).

Missing data were imputed using Multivariate Imputation by Chained Equations method [59].

Reliability: This refers to the ability of the scale to give the same results consistently if the same individual is tested at different time points. Internal consistency of the scale refers to the extent to which all items measure the same variable. We used the Cronbach’s alpha coefficient to evaluate the Arabic SOC-13 related internal consistency. Both Cronbach’s alpha and hierarchical omega were used to examine the consistency of the total SOC-13 scale and its three subscales.

## Results

Among the participants, n = 481, (38.9%) were males and n = 754, (61.1%) were females. Most participants were under 25 years of age; n = 653 (52.9%). Table 1 shows a detailed display of the basic demographic factors of the participants.

**Table 1:** Baseline demographics of the study participants

Factor	Count (n)/mean	Percentage/SD (%)
*Sex*		
Males	481	38.9
Females	754	61.1
*Age*		
18–25	653	52.9
26–33	230	18.6
34–41	149	12.1
42–49	121	9.8
50–57	65	5.3
Over 58	17	1.4
*Marital status*		
Married	414	33.5
Widow	1	0.1
Single	779	63.1
Divorced	41	33.2
*Employment*		
Employee	462	37.4
Student	558	45.2
Unemployed	214	17.3
*Region*		
Central	683	55.3
Eastern	355	28.7
Northern	42	3.4
Southern	57	4.6
Western	98	7.9
Psychiatric history	104	8.4
Current mental issues	220	17.8
On psych medications	65	5.3
Ruqia history	285	23.1
Smoking	166	13.4
Substance use	22	1.8
Psychological issues	639	51.7

### Reliability and internal consistency of the Arabic SOC-13

The reliability coefficient, Cronbach’s alpha, for the comprehensibility subscale of the Arabic SOC-13 was 0.69 (95% confidence interval CI between 0.67 and 0.72), indicative of acceptable internal consistency. None of its items has a significant impact on the overall reliability estimate. The poorest performing item in terms of internal consistency was *item 2*: “Has it happened in the past that you were surprised by the behaviour of people whom you thought you knew well?”. When item 2 was removed from the analysis, the reliability estimate improved to 0.73. The best performing item in terms of internal consistency was *item 8*: “Do you have very mixed-up feelings and ideas?”. When item 8 was removed from the analysis, the reliability estimate deteriorated to 0.57.

Similarly, the Cronbach’s alpha reliability coefficient for the manageability subscale was 0.56 (95% CI between 0.52 and 0.60), indicative of poor internal consistency. The poorest performing item in terms of internal consistency was *item 3*: “Has it happened that people whom you counted on disappointed you?”. When item 3 was removed from the analysis, the reliability estimate improved to 0.53. The best performing item in terms of internal consistency was *item 10: *“Many people—even those with a strong character—sometimes feel like sad sacks (losers) in certain situations. How often have you felt this way in the past?”. When item 10 was removed from the analysis, the reliability estimate deteriorated to 0.45.

In addition, the Cronbach’s alpha reliability coefficient for the meaningfulness subscale was 0.53 (95% CI between 0.49 and 0.57), indicative of poor internal consistency. The poorest performing item in terms of internal consistency was *item 1*: “Do you have the feeling that you don’t really care about what goes on around you?”. When item 1 was removed from the analysis, the reliability estimate improved to 0.61. The best performing item in terms of internal consistency was *item 7*: “Doing the things you do everyday is [a source of deep pleasure and satisfaction]?”. When item 7 was removed from the analysis, the reliability estimate dropped to 0.36.

Overall, the reliability coefficient for the total SOC-13 score was 0.82 (95% CI between 0.81 and 0.84), indicative of good internal consistency. All its items were of comparable impact on the overall reliability estimate. Further information is available in Additional file 1.

Split-half reliability was calculated by the *Lambda4* package in R statistical software to be 0.86, and Guttman lambda split-half reliability to be 0.83.

### Confirmatory factor analysis

We conducted a confirmatory factor analysis to guarantee that the three dimensions of comprehensibility, meaningfulness, and manageability conform satisfactorily to the Arabic adaptation of the SOC-13. We sequentially fitted one-factor, two-factor, three-factor, and four-factor models to the dataset. However, the three-factor model did not fit in a satisfactory way for the full 13-item dataset. Notably, measurement invariance analysis showed that the three-factor model failed to maintain its structure with sufficient stability across sociodemographic groups (see Tables 7, 8, and 9). The model structure did not pass configural invariance model level of testing. Expectedly it did not pass any subsequent strains imposed on the model. A fuller 4-factor structure fitted better than the models with fewer factors. An additional dimension (composed of item 8: “Do you have very mixed-up feelings and ideas?” which cross-loaded also on both the comprehensibility dimension and the fourth dimension, and item 9: “Does it happen that you have feelings inside you would rather not feel?” which loaded completely on the fourth dimension only) is required for the sample data to conform acceptably to the theoretical covariance structure. See Additional file 2 for a detailed account of the four-factor loading.

Three items were notably reversed in terms of scoring and loaded away from their corresponding dimensions in the four-factor full model. These were item 1: “Do you have the feeling that you don’t really care about what goes on around you?”, item 2: “Has it happened in the past that you were surprised by the behaviour of people whom you thought you knew well?”, and item 3: “Has it happened that people whom you counted on disappointed you?”.

Item 1: “Do you have the feeling that you don’t really care about what goes on around you?” cross-loaded equally on both factor one and factor three, whereas item 2: “Has it happened in the past that you were surprised by the behaviour of people whom you thought you knew well?” and item 3: “Has it happened that people whom you counted on disappointed you?” were lumped together loading unto factor three.

We further attempted to perform the confirmatory factor analysis of a three-factor model after removal of the three poorly performing items (item 1: “Do you have the feeling that you don’t really care about what goes on around you?”, item 2: “Has it happened in the past that you were surprised by the behaviour of people whom you thought you knew well?”, and item 3: “Has it happened that people whom you counted on disappointed you?”). This item-deleted three-factor model was substantially better than the full three-factor model in the full dataset and of even better fit than the 4-factor model. See Tables 2 and 3.

**Table 2:** Comparison of four models for the SOC-13 factor structure

Model	Chi-squared (df)	RMSEA (90% CI)	CFI	TLI	SRMR
One-factor	1110.240 (65)	0.114 (0.108–0.120)	0.944	0.933	0.077
Two-factor	760.126 (64)	0.094 (0.088–0.100)	0.963	0.955	0.067
Three-factor	614.628 (62)	0.085 (0.079–0.091)	0.971	0.963	0.060
Four-factor	537.638 (59)	0.081 (0.075–0.087)	0.975	0.966	0.057
Three-factor*	1000.292 (62)	0.111 (0.105–0.117)	0.950	0.937	0.073
Three-factor [excluding items 1,2, and 3]	267.641 (32)	0.077 (0.069–0.086)	0.985	0.979	0.053

CFI, comparative fit index; TLI, Tucker-Lewis index; RMSEA, root mean square error of approximation; CI, confidence interval; df, degrees of freedom; SRMR, standardized root-mean-square residuals

Three-factor*: The original three-dimensional structure

**Table 3:** Items to be excluded from the original SOC-13 three-facture structure to be preserved

Dimension	Item	Verbatim
Comprehensibility	2	Has it happened in the past that you were surprised by the behaviour of people whom you thought you knew well?
Manageability	3	Has it happened that people whom you counted on disappointed you?
Meaningfulness	1	Do you have the feeling that you don’t really care about what goes on around you?

For a good fit, it is preferred that the comparative fit index (CFI) > 0.95, Tucker-Lewis index (TLI) > 0.95, and root mean square error of approximation (RMSEA) < 0.06 [25]. A good fit to the dataset is shown with RMSEA value < 0.05 and an acceptable fit is when RMSEA is < 0.08 [26].

### Discriminant validity analysis

Discriminant validity is considered an index of the difference between the three underlying constructs (namely, comprehensibility, manageability, and meaningfulness). These constructs are inherently similar, therefore a strong correlation is expected between them, and, therefore, low discriminatory power. We opted to use a multiply operationalized procedure method, as portrayed in Table 4, to examine the correlation matrix of *z*-transformed scores of each of the three constructs [27].

**Table 4:** The multiply operationalized correlation of the three constructs of the SOC scale

Construct	Mean	SD	Biserial correlation	*P* value
Comprehensibility	0	0.6686	0.7125	< 0.0001
Manageability	0	0.6576	0.6999	< 0.0001
Meaningfulness	0	0.6487	0.5648	< 0.0001

### Convergent validity analysis

Convergent validity is the establishment of a significant correlation between different components of a measurement scale that assesses the same construct [28]. We examined the correlation between each of the SOC-13 items and its corresponding construct. Correlation coefficients were interpreted as: 0–0.19 = very weak; 0.20–0.39 = weak; 0.40–0.59 = moderate; 0.60–0.79 = strong; 0.80–1.0 = very strong [29].

Clearly, all items demonstrated strong convergent validity, except SOC1 and SOC2, whose convergent validity was moderate. See Table 5.

**Table 5:** Convergent validity of the SOC-13 items

Item	Construct	Biserial correlation	Interpretation	*P* value
SOC1	Meaningfulness	0.5157	Moderate	< 0.0001
SOC2	Comprehensibility	0.4966	Moderate	< 0.0001
SOC3	Manageability	0.6237	Strong	< 0.0001
SOC4	Meaningfulness	0.6903	Strong	< 0.0001
SOC5	Manageability	0.6644	Strong	< 0.0001
SOC6	Comprehensibility	0.6552	Strong	< 0.0001
SOC7	Meaningfulness	0.7253	Strong	< 0.0001
SOC8	Comprehensibility	0.7850	Strong	< 0.0001
SOC9	Comprehensibility	0.7640	Strong	< 0.0001
SOC10	Manageability	0.6915	Strong	< 0.0001
SOC11	Comprehensibility	0.6915	Strong	< 0.0001
SOC12	Meaningfulness	0.6636	Strong	< 0.0001
SOC13	Manageability	0.6508	Strong	< 0.0001

### Effect of demographic and clinical factors on SOC-13 score

**Fig. 1:**
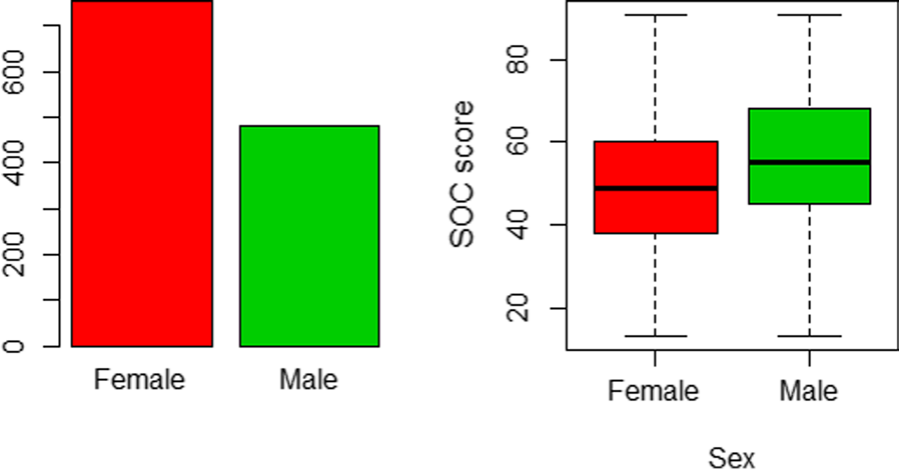
Sex distribution of SOC-13 score among participants. Figure demonstrates that females were (n = 754, 61.1%) of the sample, compared to males who were (n = 481, 38.9%). However, males scored substantially better than females. The mean SOC-13 score in men was 56.5 points, compared to the mean score of SOC-13 among women that was 49.3 points. This difference was statistically significant (t = 7.782, *P* < 0.0001)

**Fig. 2:**
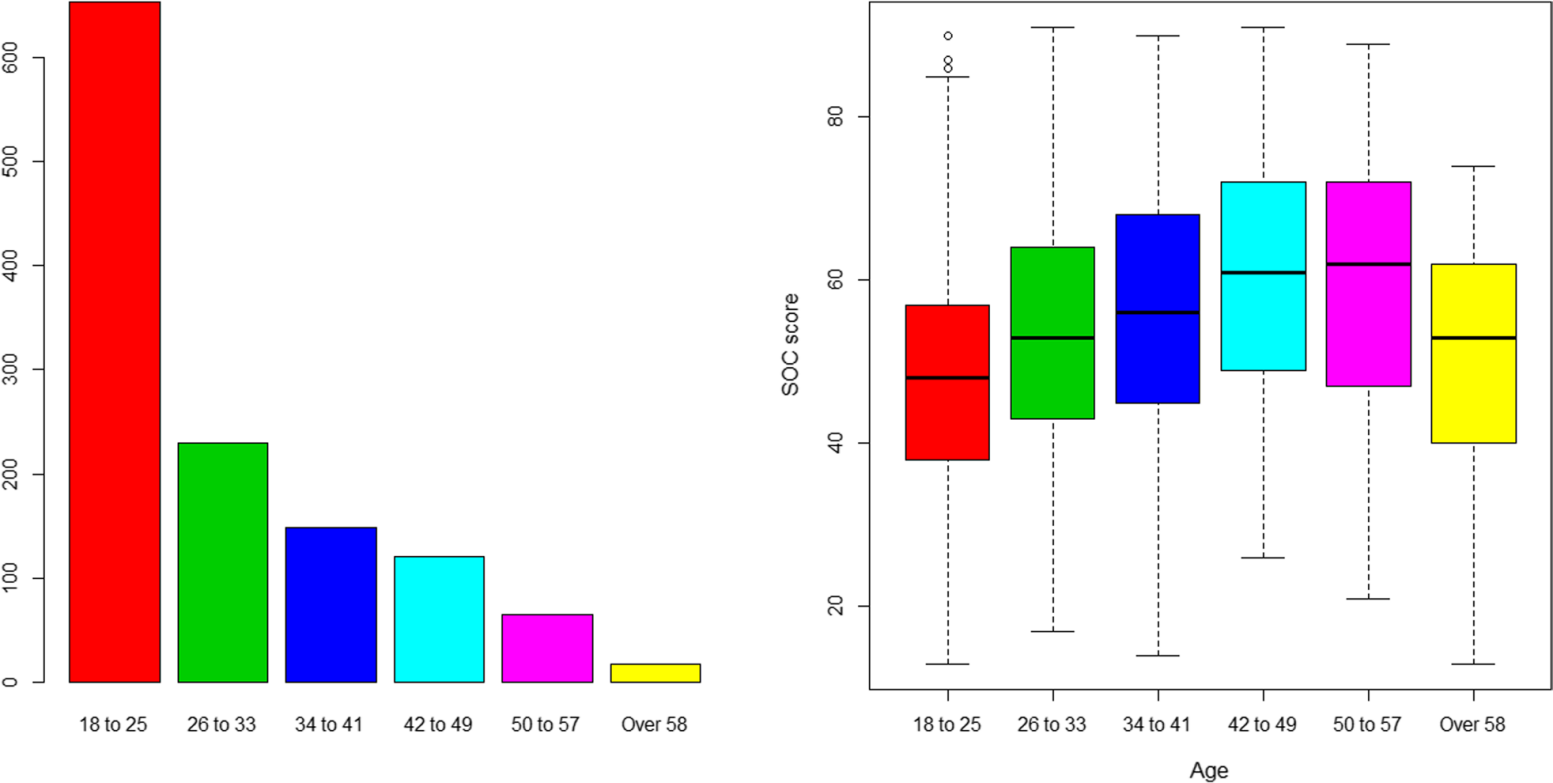
Age distribution among the participants and the SOC-13 score. Figure demonstrates that the majority of the participants were under 25 (n = 653, 52.9%). The difference in terms of age distribution was also statistically significant (F = 20.92, *P* < 0.0001). The 42-to-49 class scored best in terms of SOC-13 (mean = 60.8), whereas the over 85 scored the lowest (mean = 48.2)

**Fig. 3:**
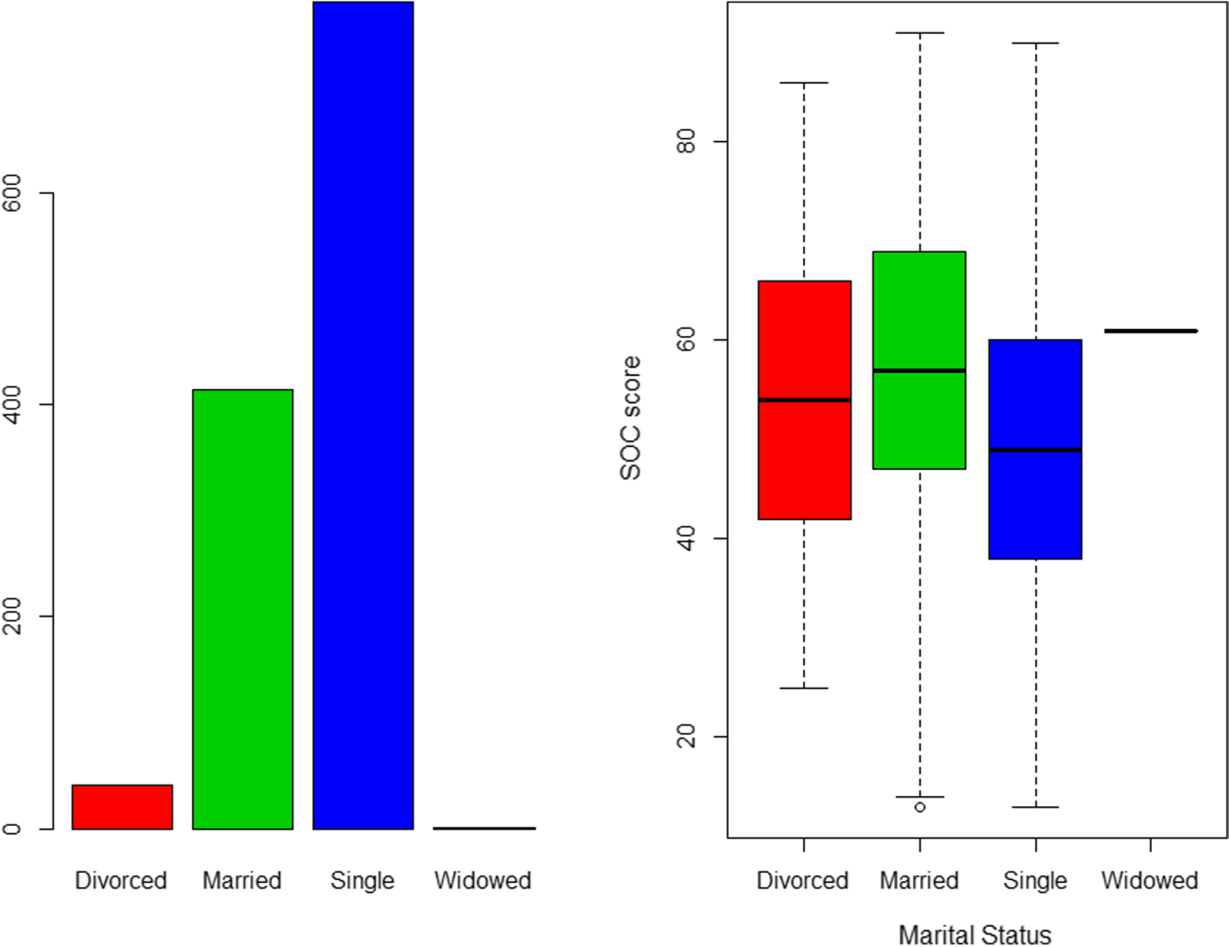
Distribution of marital status and SOC-13 score among the participants. Figure demonstrates that most participants (n = 779, 63.1%) were single. However, single participants scored the least SOC-13 (mean = 49.4) compared to the married (mean = 57.0). This was statistically significant (F = 21.51, *P* < 0.0001)

**Fig. 4:**
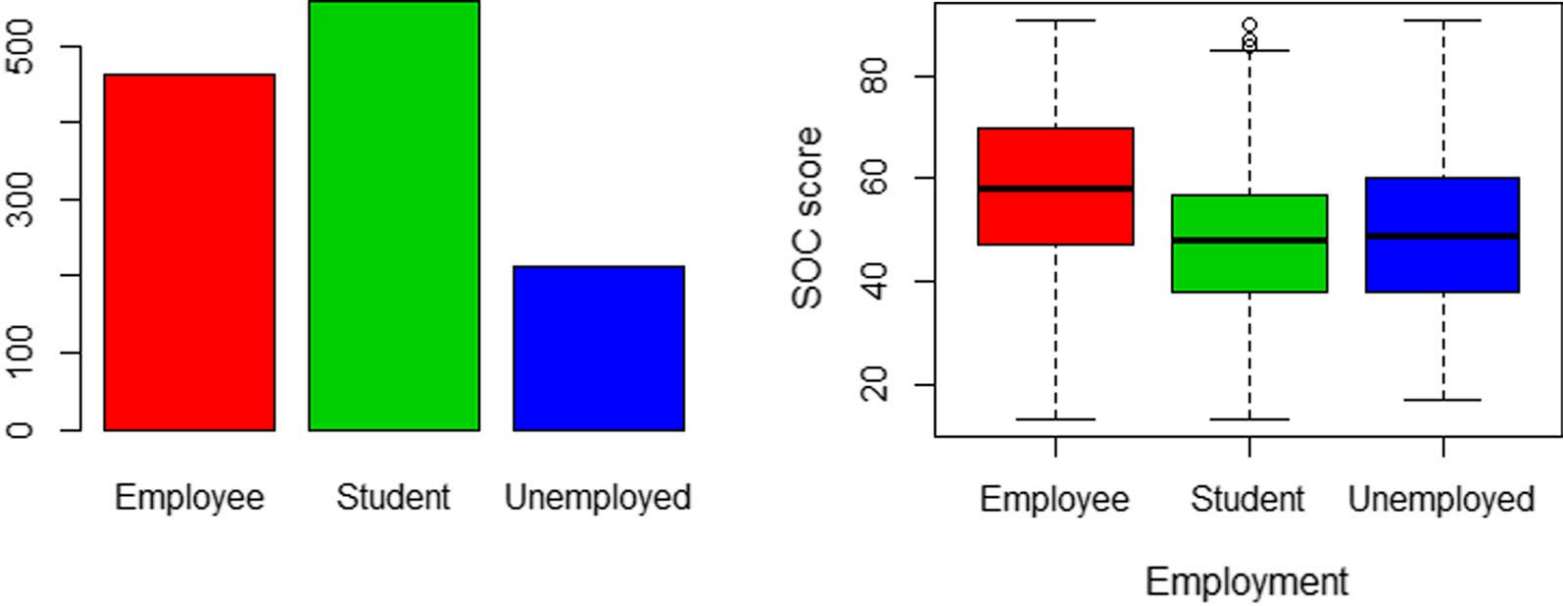
Employment status and SOC-13 score. Figure shows that students (n = 558, 45.2%) constituted the majority class, followed by the employed (n = 462, 37.4%). However, students scored the least SOC-13 (mean = 48.4) compared to the employees (mean = 57.8). This difference was statistically significant (F = 50.46, *P* < 0.0001)

**Fig. 5:**
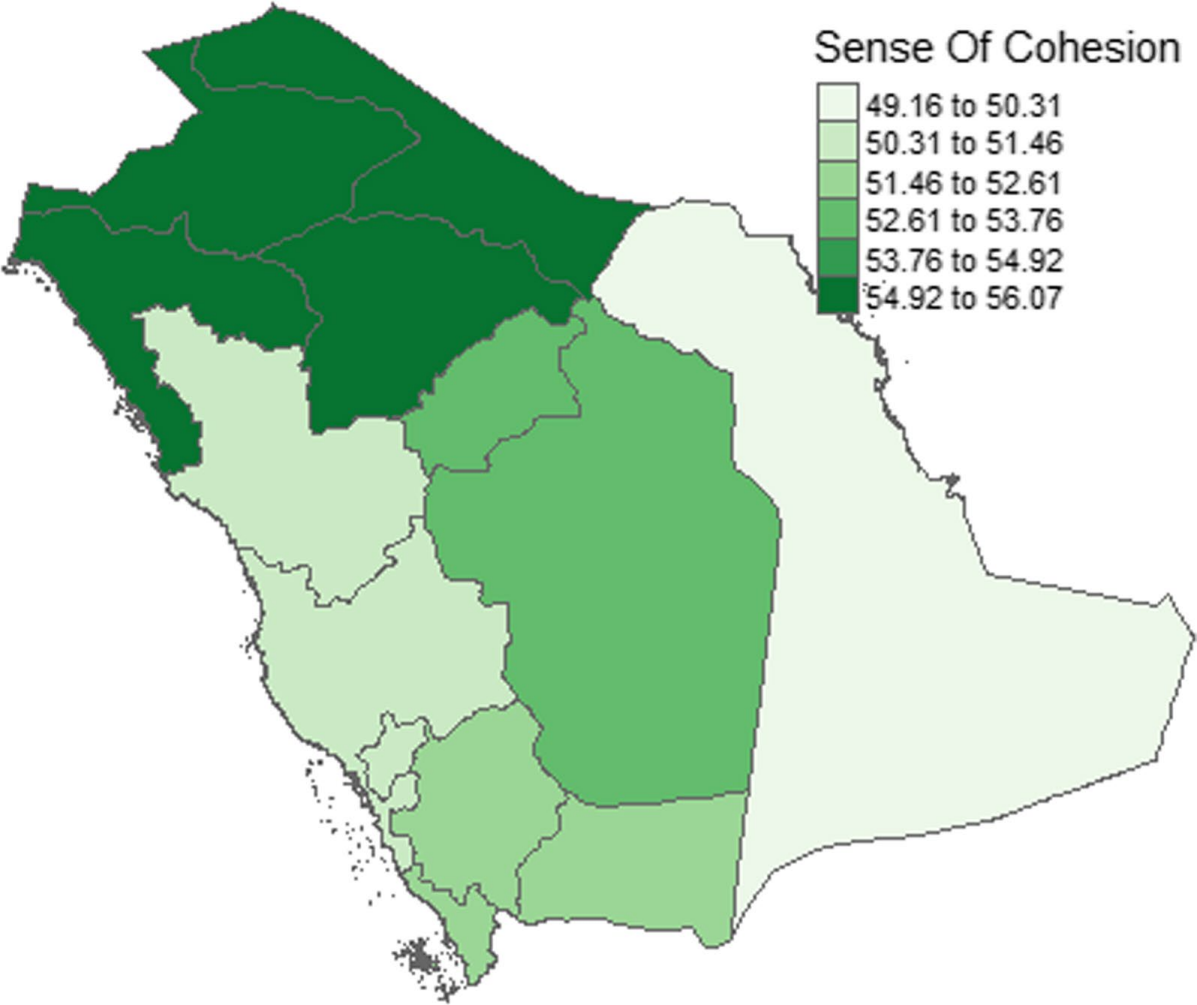
Distribution of OC-13 scores by geographic region

**Fig. 6:**
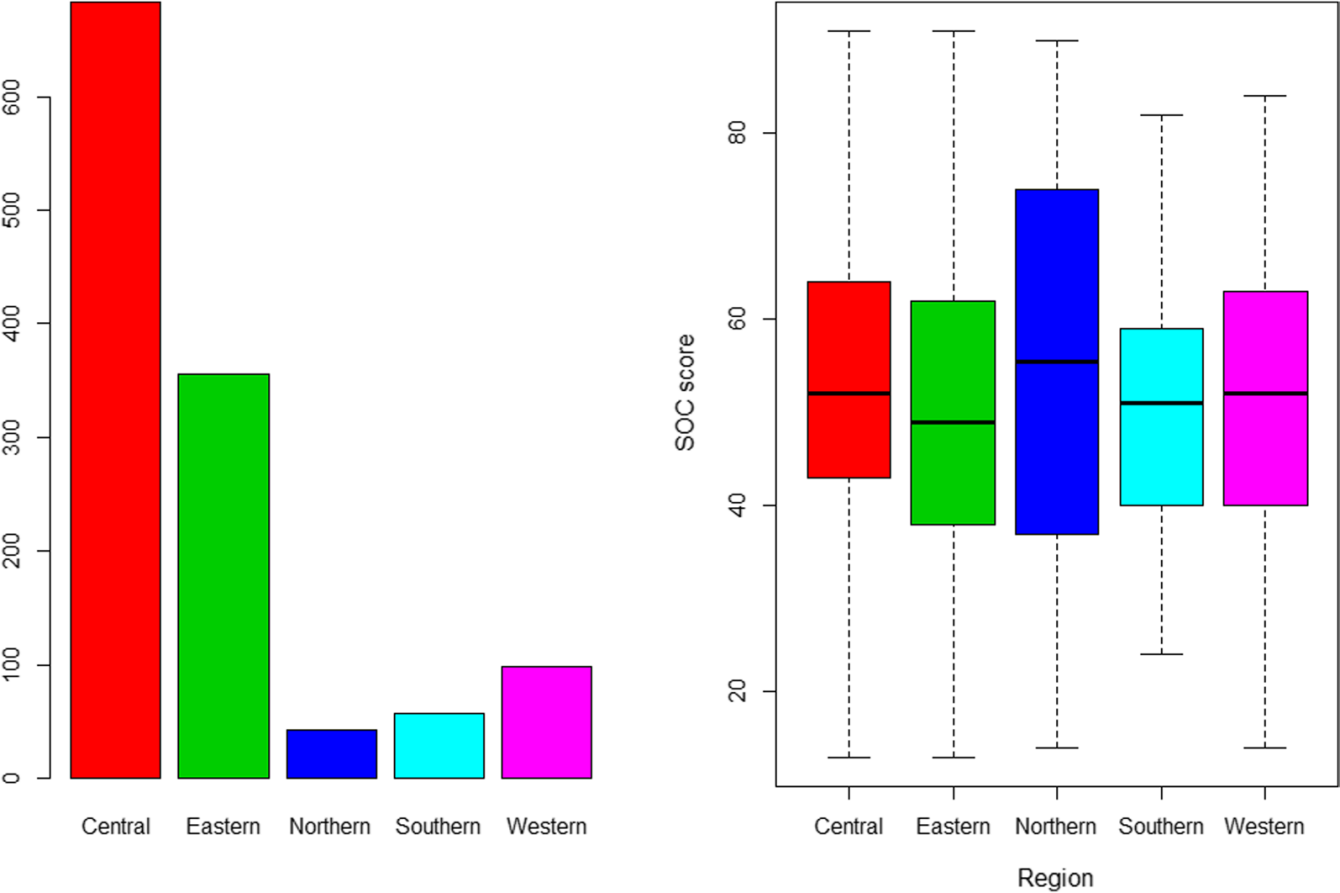
Distribution of geographic regions among the participants and their SOC-13 score. Figure demonstrates that most of the participants (n = 683, 55.3%) were from the central region. However, the highest SOC-13 score was observed in the Northern Region (mean = 55.7), whereas the least score was from the Eastern Region (mean = 49.5). This difference was statistically significant also (F = 3.908, *P* = 0.0037)

The following Table 6 and the Figs. 1, 2, 3, 4, 5 and 6 provide a detailed account of the effect of background factors on the sense of cohesion as measured by the SOC-13 scale.

**Table 6:** Effect of baseline demographics of the study participants on their SOC-13 score

Factor	Mean of SOC-13 score	T test (F test) value	*P* value
*Sex*			
Males	56.5	t = 7.7822	1.803 × 10^–14^
Females	49.3		
*Age*			
18–25	48.3	F = 20.92	< 2 × 10^–16^
26–33	53.9		
34–41	55.8		
42–49	60.8		
50–57	59.2		
Over 58	48.2		
*Marital status*			
Married	57.0	F = 21.51	1.39 × 10^–13^
Widow	61.0		
Single	49.4		
Divorced	53.3		
*Employment*			
Employee	57.8	F = 50.46	< 2 × 10^–16^
Student	48.4		
Unemployed	49.3		
*Region*			
Central	53.3	F = 3.908	0.00369
Eastern	49.5		
Northern	55.7		
Southern	51.6		
Western	51.1		
Psychiatric history	Yes = 47.3	3.1289	0.002195
	No = 52.5		
Current mental issues	Yes = 39.8	15.121	< 2 × 10^–16^
	No = 54.7		
On psych medications	Yes = 46.0	3.211	0.00198
	No = 52.4		
Ruqia history	Yes = 46.9	6.3599	4.757 × 10^−10^
	No = 53.6		
Smoking	Yes = 51.8	0.2093	0.8344
	No = 52.1		
Substance use	Yes = 45.0	2.2371	0.0358
	No = 52.2		
Psychological issues	Yes = 46.4	13.724	< 2 × 10^–16^
	No = 58.1

The mean overall SOC-13 total score in our sample was 52.1 (SD = 16.1), ranging between 13 and 91. The median SOC-13 score was 51.

Regarding gender distribution, males scored substantially better than females. The mean SOC-13 score in men was 56.5 points, compared to the mean score of SOC-13 among women that was 49.3 points. This difference was statistically significant (t = 7.782, *P* < 0.0001). See Figure.

The difference in terms of age distribution was also statistically significant (F = 20.92, *P* < 0.0001). The 42-to-49 class scored best in terms of SOC-13 (mean = 60.8), whereas over 85 scored the lowest (mean = 48.2). See Fig. 2 (Table 7).

**Table 7:** Measurement invariance analysis for males and females based on the theoretical three-factor model

Factor	χ^2^ (df)	Δ χ^2^ (Δdf)	Δ χ^2^ *P*	CFI	Δ CFI	RMSEA	ΔRMSEA
Base: men	430 (62)			0.956		0.111	
Base: women	710 (62)			0.933		0.118	
Configural invariance gender	1141 (124)	431 (62)	< 0.0001	0.944	0.011	0.115	0.003
Metric invariance gender	1230 (134)	89 (10)	< 0.0001	0.940	0.004	0.115	0

Regarding marital status, as shown in Fig. 3, single participants scored the least SOC-13 (mean = 49.4) compared to the married (mean = 57.0). This was statistically significant (F = 21.51, *P* < 0.0001).

Regarding employment status, students scored the least SOC-13 (mean = 48.4) compared to the employees (mean = 57.8). This difference was statistically significant (F = 50.46, *P* < 0.0001). See Fig. 4 (Table 8).

**Table 8:** Measurement invariance analysis for different age categories based on the theoretical three-factor model

Factor	χ^2^ (df)	Δ χ^2^ (Δdf)	Δ χ^2^ *P*	CFI	Δ CFI	RMSEA	ΔRMSEA
Base: 18-to-25	709 (62)			0.901		0.127	
Base: 26-to-33	225 (62)			0.959		0.107	
Base: 34-to-41	185 (62)			0.967		0.116	
Base: 42-to-49	181 (62)			0.953		0.127	
Configural invariance: age	1557 (372)	868 (310)	< 0.0001	0.942		0.125	
Metric invariance: age	2051 (422)	494 (50)	< 0.0001	0.920	0.022	0.137	0.012

Regarding the Kingdom region of the participants, see Fig. 5, the highest SOC-13 score was observed in the Northern Region (mean = 55.7), whereas the least score was in the Eastern Region (mean = 49.5). This difference was statistically significant also (F = 3.908, *P* = 0.0037). See Fig. 1 below, and See Fig. 6.

**Fig. 7:**
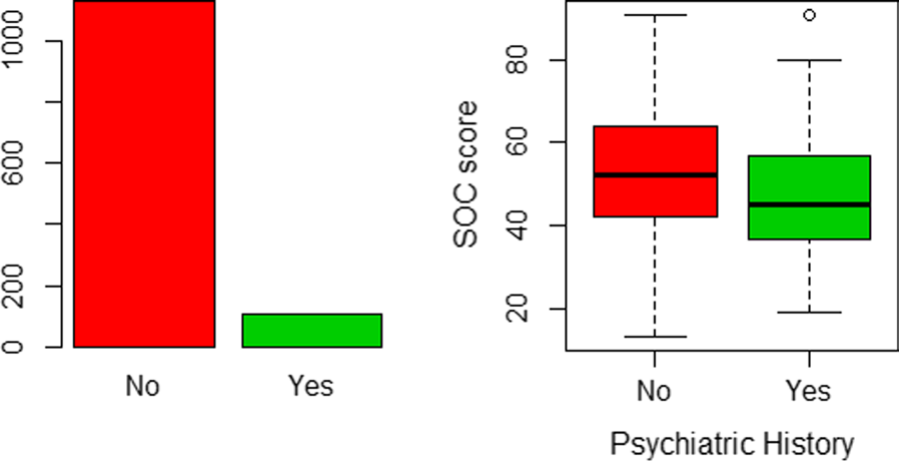
Effect of psychiatric history on SOC-13 score. Figure shows that only a minority (n = 104, 8.4%) reported history of psychiatric illness. They scored significantly lower (mean SOC-13 = 47.3) than those with no psychiatric history (mean = 52.5). *P* = 0.002195

Presence of psychiatric history was associated with a mean SOC-13 score of 47.3 (substantially less than for participants who reported no psychiatric history whose mean SOC-13 score was 52.5, t = 3.1289, *P* = 0.002195). See Fig. 7 below.

## Discussion

We found the overall internal consistency for the SOC-13 measure to be 0.82, quite high indeed and allows reliable decision making based on the Arabic SOC-13 scale [30]. This corroborates the figure of 0.75 found by the Al-Yateem et al. [2020] [12] and is even closer to the 0.83 reliability estimate found by Stern et al. [2019] [15]. Several international studies have provided estimates for the reliability of the SOC-13 between 0.70 and 0.93 [31–34]. However, the reliability for the subscales was less impressive in our findings. The Cronbach’s alpha estimates were 0.69, 0.56, and 0.53 for comprehensibility, manageability, and meaningfulness subscales, respectively. The corresponding figures in the Slovenian evaluation were far better for the reliability of the subdimensions, namely, 0.66, 0.69, and 0.79, respectively [15]. However, the Slovenian sample was far more homogeneous than our sample, as they recruited patients with Multiple Sclerosis. That homogeneity could have overestimated the reliability scores (Table 9).

**Table 9:** Measurement invariance analysis for marital status, employment, and psychiatric history categories based on the theoretical three-factor model

Factor	χ^2^ (df)	Δ χ^2^ (Δdf)	Δ χ^2^ *P*	CFI	Δ CFI	RMSEA	ΔRMSEA
Configural invariance: marital	1234 (186)			0.948		0.117	
Metric invariance: marital	1631 (206)	403 (20)	< 0.0001	0.929	0.019	0.130	0.013
Configural invariance: employment	1265 (186)			0.942		0.119	
Metric invariance: employment	1431 (206)	166 (20)	< 0.0001	0.934	0.008	0.120	0.001
Configural invariance: psych history	1179 (124)			0.945		0.117	
Metric invariance: psych history	1246 (134)	65 (10)	< 0.0001	0.942	0.003	0.116	0.001

Our split-half reliability and Guttmen lambda estimates were 0.86 and 0.83, respectively. They were far better than the estimates reported by *Rajesh *et al*.* [2016] [16], of 0.71 and 0.70. This may indicate better stability for the Arabic SOC-13, or may be an artefact of the large sample size in our study.

Clearly, the three first items SOC1, SOC2, and SOC3 were negatively impacted on the reliability of the subscales. Many previous studies pointed out these items as problematic [9]. They stir strong feelings of the sort of ‘how such a question could be asked?’ which could negatively affect the respondents’ choice.

Those three items were all reversed in terms of scoring. This is known to be confusing to the respondents and could have affected our participants’ interpretation of the item verbatim [35]. In general, the use of reversely worded items serves to remove ‘acquiescence bias’. Acquiescence bias refers to the tendency of the participant to automatically tick a certain item repeatedly without comprehending its actual content [36]. However, the inclusion of such reversely phrased items was proved to negatively affect the factor structure of the overall scale [37]. The fact that the removal of the three reversely phrased items vastly improved the factor = structure of the SOC-13 Arabic scale can be regarded as supportive to the notion prosed by *DiStefano and Motl* [2006] [38]. They suggested that reversely worded items tend to move away from the theoretical factor structure as they measure a ‘method dimension’ that was not originally designed by the researcher. Notably, the three reversely phrased items in our sample loaded away from their corresponding factors. This could be the reason for the recently proposed notion that although reversely articulated questions may guard against acquiescence bias in collective scale scoring, they would not be expected to control acquiescence bias in the underlying latent factor structure [39].

Item 1: “Do you have the feeling that you don’t really care about what goes on around you?” was designed to load on the meaningfulness dimension. This is the motivational dimension concerned with commitment, dedication, the will for energy spending, and emotional meaning of being able to solve every-day problems [7]. Item 1 clearly reflects a sense of indifference to internal or external challenges. It loaded on manageability and comprehensibility dimensions. In our participants, they were stuck more in the cognitive-behavioural aspects rather than the motivational facets of the “Do you have the feeling that you don’t really care about what goes on around you?” question. The reason is difficult to clarify.

In the study conducted by [14], item 1 (in addition to item 5) was noted to have a poor fit to the overall factor structure of the SOC-13. They regarded item 1 as a hurdle against satisfactory internal scale validity and suggested its permanent drop from the SOC-13. They found similar results in another survey of morbidly obsessed individuals [40].

Similarly, item 2: “Has it happened in the past that you were surprised by the behaviour of people whom you thought you knew well?” and item 3: “Has it happened that people whom you counted on disappointed you?” did not conform to the predesigned respective facets of cognitive and behavioural dimensions. They may measure an interactionist dimension, or a trust-building ability rather than a cognitive ‘surprise’ or behavioural ‘disappointment’ as suggested by *Eriksson *et al*.* [2017] [7] classification. It could well be said that they measure a ‘methods’ dimension’ given their reversely worded verbatim [38]. The complexity of how these items interact requires well-designed exploratory studies.

A closer examination of the three problematic items could reveal a potential difference between them. Item 1 is a type of ‘negation item’, whereas both item 2 and item 3 are ‘polar opposite items’ [41]. All these items, negation and polar opposite phrases, were shown to discourage the so-called “yea tendency”, i.e., giving a target-unrelated response to the item, thereby shaking the underlying theoretical factor structure [42]. It may well be that the structure of these items is the main reason for their unfavourable psychometric characteristics rather than their content.

Item 2 “Has it happened in the past that you were surprised by the behaviour of people whom you thought you knew well?” was particularly tricky in numerous cultural adaptations of the SOC-13 scale, including Peruvian [43], Italian [44], and Dutch [45]. Item 2 was regarded as troublesome in a recent survey of Swedish subjects, and the SOC-29 was substantially better in terms of factor structure when it was removed [46].

Clearly, item 2 bears a close relation to the concept of ‘predictability’. This predictability concept constituted one of the foci for criticism of the SOC-13 scale. Many authors argued that for a high sense of coherence there is no need for life to be predictable to the individual [47]. Conversely, unpredictability could provide a pleasant tastefulness to a healthy life. This was proposed as the primary reason for the poor correlation of SOC-13 and physical health measures [48].

Our results demonstrate that for the Arabic SOC-13 to have a good fit to the underlying three-factor structure, three items require removal. Namely, item 1 needs to be removed from the meaningfulness dimension, item 2 needs to be deleted from the comprehensibility dimension, and item 3 requires elimination from the manageability dimension. Notably, these very items performed poorly in terms of internal consistency. It is unclear why the exclusion of these questions results in the improvement of reliability and model structure. One speculative explanation may be the reverse nature of the questions that merit further explanation of the social desirability bias.

We found the mean SOC-13 score to be 52.1, which was clearly lower than the mean reported by *Ferguson *et al*.* [2015] [17] of 67.5. This may be difficult to explain, but the sample in the *Ferguson *et al*.* [2015] [17] investigation consisted of homogeneous pregnant women, who were more likely to be physically and emotionally more stable (and, hence, of a higher sense of coherence) than our heterogeneous sample.

In terms of the impact of demographic variables on the sense of coherence, we demonstrated that it improves with time, till the age of 42. It then stabilizes till older age and drops in the ninth decade. This nearly agrees with the initial assumptions proposed by *Antonovsky* [1987] [2]. He suggested that the sense of coherence continues to grow until the fourth decade of life with reasonable stability until the retirement age, before it declines. However, several researchers demonstrated that the sense of coherence does not cease developing throughout life [49–51]. Evidence from large-scale studies was consistent that the sense of coherence increases with age as life experiences accumulate [52]. Recent studies corroborate the notion that age does not substantially influence the sense of coherence, particularly in individuals over 65 years of age [53].

Expectedly, we found that subjects who reported mental health difficulties were of substantially lower levels in terms of sense of coherence than people who did not report psychological ill health. This agrees with the results of several recent investigations that confirmed that the sense of coherence can reliably predict mental health across a range of populations and age groups [54–56]. One likely explanation is the established inverse relationship between mental health difficulties and quality of life [13]. One further explanation could be the limitations posed by ill mental health on physical activity and the sense of optimism [57].

We also demonstrated a significant impact of marital status on the sense of coherence score. Many studies were consistent in terms of the effect of family relationship on the sense of coherence [58].

In our exploration, we found Northern and central areas in Saudi Arabia to be exceedingly better in terms of sense of coherence than Eastern and Western parts. This may be difficult to explain fully. Urban areas were shown in many studies to be more resourceful in terms of mental health facilities that were reflected in better emotional well-being [57]. Clearly, further research is required to explain the factors that cause such sense of coherence related disparity in Saudi Arabia to occur.

Further research should attempt rewording those three items and set up focus groups to explore how respondents felt about these specific items. It will be worthwhile examining the factor structure of the Arabic SOC-13 scale with the reversely worded items included in their nonreversed version. It is hoped that such a way of presenting the SOC-13 may improve the reliability estimates and the overall psychometric characteristics.

A focus on the cognitive-behavioural underpinnings of item 1: “Do you have the feeling that you don’t really care about what goes on around you?” Among Arabic-speaking subjects, it deserves focused and dedicated exploration via preferably qualitative methods.

Moreover, item 2: “Has it happened in the past that you were surprised by the behaviour of people whom you thought you knew well?” and item 3: “Has it happened that people whom you counted on disappointed you?” were noted to reflect something other than cognitive behavioural dimensions. Their exact latent content can be elucidated via large-scale exploratory studies that could also use qualitative design.

Future research in Saudi Arabia should attempt to clarify regional differences in terms of the sense of coherence. Factors such as urbanicity, resourcefulness, and resource accessibility should be explored as possible moderators in terms of sense of coherence and emotional well-being.

Moreover, the effect of homogeneity of the underlying study population on the reliability score deserves focused research in the future. A comparison should be made between participants with common characteristics, such as common endocrinological or neurological conditions (e.g., diabetes or multiple sclerosis), and participants with no such common characteristics.

The current study has several strengths. The sample size was large. However, one substantial imitation was recruitment of under 25 majority. That may not represent the demographics of Saudi population; therefore, care is needed when generalizing our results. Moreover, the cross-sectional and online design would not elucidate bidirectional associations or selection bias, as computer and internet literacy is an important filter in selecting participants.

## Supplementary Information

**Additional file 1.** Details of reliability coefficient for the total SOC-13 score and each of the three sub-scores and each of the thirteen individual items.

**Additional file 2.** Detailed account of the four-factor loading for the Arabic SOC-13 data.

**Additional file 3.** Full dataset for the Arabic SOC-13 responses among the participating individuals.

## Data Availability

Full dataset is included as a Additional file 3 titles “SOC13-Data”.

## Abbreviations

SOC: Sense of coherence
CFA: Confirmatory factor analysis
RMSEA: Root mean square error of approximation
CFI: Comparative fit index
GFI: Goodness-of-fit index
RMR: Root mean square residual
SRMR: Standardized root mean square residual
