# Supplementary material for: Validation of the factor structure and psychometric characteristics of the Arabic adaptation of the sense of coherence SOC-13 scale: a confirmatory factor analysis
Source: BMC Psychol. 2022 May 3;10:115. doi: 10.1186/s40359-022-00826-4 (PMC9066761; doi:10.1186/s40359-022-00826-4)
Supplement: Supplementary file 1 — Additional file 1. Details of reliability coefficient for the total SOC-13 score and each of the three sub-scores and each of the thirteen individual items. [file 40359_2022_826_MOESM1_ESM.docx]

**Validation of the Factor Structure and Psychometric Characteristics of the Arabic Adaptation of the Senses of Coherence SOC-13 Scale: a Confirmatory Factor Analysis**

# Authors:

Dr Fatimah Sayer Alharbi,

Assistant Professor in Mental Health Psychology, Nora University

Abdulaziz I Aljemaiah,

Senior Clinical Psychologist, Armed Forces Centre for Psychiatric Care, Taif, Saudi Arabia

Dr Mugtaba Osman,

Consultant Psychiatrist, Armed Forces Centre for Psychiatric Care, Taif, Saudi Arabia

Corresponding Author: Dr Mugtaba Osman, Email: [Mugtaba.osman@ucdconnect.ie](mailto:Mugtaba.osman@ucdconnect.ie), Address: Armed Forces Centre for Psychiatric Care, Prince Mansour Military Hospital, Al-Matar Street, Al-Faiysaliyah District, Taif, Saudi Arabia

Reliability analysis for comprehensibility subscale

| raw α | std. α | G6(smc) | average r | S/N | ase | mean | sd | median-r |
| --- | --- | --- | --- | --- | --- | --- | --- | --- |
| 0.7 | 0.69 | 0.68 | 0.31 | 2.2 | 0.013 | 3.6 | 1.5 | 0.33 |

- raw α: alpha based upon the covariances
- std. α: The standardized alpha based upon the correlations
- G6(smc): Guttman's Lambda 6 reliability
- smc = (the squared multiple correlation)
- Average r: The average interitem correlation
- S/N: Signal/Noise ratio (the ratio of reliable variance to unreliable variance)
- Ase: alpha standard error
- mean: For data matrices, the mean of the scale formed by summing the items
- sd: For data matrices, the standard deviation of the total score
- r: The correlation of each item with the total score (not corrected for item overlap)
- median-r: The median interitem correlation

| lower 95% confidence | alpha | upper 95% confidence |
| --- | --- | --- |
| 0.67 | 0.7 | 0.72 |

Reliability if an item is dropped:

|  | raw α | std. α | G6(smc) | average r | S/N | α se | var.r | med.r |
| --- | --- | --- | --- | --- | --- | --- | --- | --- |
| dat.SOC2 | 0.73 | 0.73 | 0.70 | 0.40 | 2.7 | 0.012 | 0.017 | 0.35 |
| dat.SOC6 | 0.66 | 0.65 | 0.63 | 0.32 | 1.8 | 0.016 | 0.037 | 0.28 |
| dat.SOC8 | 0.57 | 0.56 | 0.51 | 0.24 | 1.3 | 0.020 | 0.012 | 0.25 |
| dat.SOC9 | 0.58 | 0.58 | 0.52 | 0.26 | 1.4 | 0.019 | 0.013 | 0.26 |
| dat.SOC11 | 0.66 | 0.66 | 0.64 | 0.32 | 1.9 | 0.015 | 0.039 | 0.28 |

Item statistics

|  | n | raw r | std r | r cor | r drop | mean | sd |
| --- | --- | --- | --- | --- | --- | --- | --- |
| dat.SOC2 | 1235 | 0.48 | 0.50 | 0.26 | 0.22 | 3.5 | 2.1 |
| dat.SOC6 | 1235 | 0.66 | 0.66 | 0.51 | 0.43 | 4.2 | 2.2 |
| dat.SOC8 | 1235 | 0.79 | 0.79 | 0.77 | 0.62 | 3.5 | 2.3 |
| dat.SOC9 | 1235 | 0.77 | 0.76 | 0.73 | 0.59 | 3.2 | 2.3 |
| dat.SOC11 | 1235 | 0.64 | 0.64 | 0.48 | 0.41 | 3.6 | 2.2 |

Non missing response frequency for each item

|  | 1 | 2 | 3 | 4 | 5 | 6 | 7 | miss |
| --- | --- | --- | --- | --- | --- | --- | --- | --- |
| dat.SOC2 | 0.28 | 0.08 | 0.14 | 0.17 | 0.11 | 0.11 | 0.11 | 0 |
| dat.SOC6 | 0.21 | 0.06 | 0.13 | 0.15 | 0.09 | 0.10 | 0.26 | 0 |
| dat.SOC8 | 0.36 | 0.07 | 0.11 | 0.12 | 0.08 | 0.08 | 0.18 | 0 |
| dat.SOC9 | 0.42 | 0.08 | 0.11 | 0.09 | 0.06 | 0.09 | 0.15 | 0 |
| dat.SOC11 | 0.31 | 0.08 | 0.09 | 0.16 | 0.12 | 0.06 | 0.18 | 0 |

Reliability analysis for manageability subscale

| raw α | std. α | G6(smc) | average r | S/N | ase | mean | sd | median-r |
| --- | --- | --- | --- | --- | --- | --- | --- | --- |
| 0.56 | 0.56 | 0.5 | 0.24 | 1.3 | 0.02 | 3.9 | 1.4 | 0.27 |

| lower 95% confidence | alpha | upper 95% confidence |
| --- | --- | --- |
| 0.52 | 0.56 | 0.6 |

Reliability if an item is dropped:

|  | raw α | std. α | G6(smc) | average r | S/N | α se | var.r | med.r |
| --- | --- | --- | --- | --- | --- | --- | --- | --- |
| dat.SOC3 | 0.53 | 0.53 | 0.43 | 0.27 | 1.13 | 0.023 | 0.00014 | 0.28 |
| dat.SOC5 | 0.48 | 0.48 | 0.39 | 0.24 | 0.93 | 0.026 | 0.00541 | 0.28 |
| dat.SOC10 | 0.45 | 0.45 | 0.36 | 0.21 | 0.81 | 0.027 | 0.00406 | 0.21 |
| dat.SOC13 | 0.49 | 0.50 | 0.40 | 0.25 | 1.00 | 0.025 | 0.00133 | 0.26 |

Item statistics

|  | n | raw r | std r | r cor | r drop | mean | sd |
| --- | --- | --- | --- | --- | --- | --- | --- |
| dat.SOC3 | 1235 | 0.61 | 0.62 | 0.40 | 0.29 | 3.8 | 2.1 |
| dat.SOC5 | 1235 | 0.69 | 0.66 | 0.47 | 0.36 | 4.3 | 2.4 |
| dat.SOC10 | 1235 | 0.67 | 0.69 | 0.53 | 0.39 | 3.6 | 2.0 |
| dat.SOC13 | 1235 | 0.66 | 0.65 | 0.45 | 0.34 | 3.9 | 2.2 |

Non missing response frequency for each item

|  | 1 | 2 | 3 | 4 | 5 | 6 | 7 | miss |
| --- | --- | --- | --- | --- | --- | --- | --- | --- |
| dat.SOC3 | 0.22 | 0.08 | 0.16 | 0.17 | 0.11 | 0.11 | 0.15 | 0 |
| dat.SOC5 | 0.23 | 0.07 | 0.09 | 0.11 | 0.09 | 0.09 | 0.31 | 0 |
| dat.SOC10 | 0.24 | 0.08 | 0.15 | 0.18 | 0.11 | 0.13 | 0.10 | 0 |
| dat.SOC13 | 0.25 | 0.08 | 0.13 | 0.14 | 0.09 | 0.11 | 0.20 | 0 |

Reliability analysis for meaningfulness subscale

| raw α | std. α | G6(smc) | average r | S/N | ase | mean | sd | median-r |
| --- | --- | --- | --- | --- | --- | --- | --- | --- |
| 0.53 | 0.54 | 0.49 | 0.23 | 1.2 | 0.022 | 4.6 | 1.4 | 0.24 |

| lower 95% confidence | alpha | upper 95% confidence |
| --- | --- | --- |
| 0.49 | 0.53 | 0.57 |

Reliability if an item is dropped:

|  | raw α | std. α | G6(smc) | average r | S/N | α se | var.r | med.r |
| --- | --- | --- | --- | --- | --- | --- | --- | --- |
| dat.SOC1 | 0.61 | 0.61 | 0.51 | 0.34 | 1.56 | 0.019 | 0.003 | 0.313 |
| dat.SOC4 | 0.41 | 0.42 | 0.34 | 0.19 | 0.71 | 0.029 | 0.011 | 0.167 |
| dat.SOC7 | 0.36 | 0.37 | 0.30 | 0.16 | 0.58 | 0.032 | 0.018 | 0.099 |
| dat.SOC12 | 0.44 | 0.45 | 0.39 | 0.21 | 0.82 | 0.028 | 0.030 | 0.167 |

Item statistics

|  | n | raw r | std r | r cor | r drop | mean | sd |
| --- | --- | --- | --- | --- | --- | --- | --- |
| dat.SOC1 | 1235 | 0.54 | 0.52 | 0.20 | 0.15 | 4.5 | 2.3 |
| dat.SOC4 | 1235 | 0.68 | 0.69 | 0.55 | 0.38 | 4.8 | 2.1 |
| dat.SOC7 | 1235 | 0.70 | 0.73 | 0.60 | 0.44 | 5.1 | 2.0 |
| dat.SOC12 | 1235 | 0.67 | 0.66 | 0.47 | 0.34 | 4.1 | 2.2 |

Non missing response frequency for each item

|  | 1 | 2 | 3 | 4 | 5 | 6 | 7 | miss |
| --- | --- | --- | --- | --- | --- | --- | --- | --- |
| dat.SOC1 | 0.19 | 0.04 | 0.08 | 0.16 | 0.11 | 0.10 | 0.32 | 0 |
| dat.SOC4 | 0.13 | 0.05 | 0.07 | 0.14 | 0.14 | 0.13 | 0.34 | 0 |
| dat.SOC7 | 0.10 | 0.02 | 0.07 | 0.18 | 0.15 | 0.13 | 0.35 | 0 |
| dat.SOC12 | 0.20 | 0.09 | 0.14 | 0.15 | 0.09 | 0.08 | 0.25 | 0 |

Reliability analysis for total SOC-13 score

| raw α | std. α | G6(smc) | average r | S/N | ase | mean | sd | median-r |
| --- | --- | --- | --- | --- | --- | --- | --- | --- |
| 0.82 | 0.82 | 0.83 | 0.26 | 4.6 | 0.0074 | 4 | 1.2 | 0.27 |

| lower 95% confidence | alpha | upper 95% confidence |
| --- | --- | --- |
| 0.81 | 0.82 | 0.84 |

Reliability if an item is dropped:

|  | raw α | std. α | G6(smc) | average r | S/N | α se | var.r | med.r |
| --- | --- | --- | --- | --- | --- | --- | --- | --- |
| dat.SOC2 | 0.82 | 0.82 | 0.82 | 0.27 | 4.5 | 0.0074 | 0.013 | 0.28 |
| dat.SOC6 | 0.81 | 0.80 | 0.82 | 0.26 | 4.1 | 0.0080 | 0.015 | 0.26 |
| dat.SOC8 | 0.79 | 0.79 | 0.80 | 0.24 | 3.8 | 0.0086 | 0.012 | 0.25 |
| dat.SOC9 | 0.80 | 0.79 | 0.80 | 0.24 | 3.9 | 0.0085 | 0.012 | 0.25 |
| dat.SOC11 | 0.81 | 0.81 | 0.82 | 0.26 | 4.2 | 0.0079 | 0.015 | 0.27 |
| dat.SOC3 | 0.82 | 0.82 | 0.82 | 0.27 | 4.4 | 0.0075 | 0.014 | 0.28 |
| dat.SOC5 | 0.81 | 0.80 | 0.82 | 0.26 | 4.1 | 0.0080 | 0.015 | 0.26 |
| dat.SOC10 | 0.81 | 0.81 | 0.82 | 0.26 | 4.2 | 0.0080 | 0.016 | 0.26 |
| dat.SOC13 | 0.80 | 0.80 | 0.81 | 0.25 | 4.0 | 0.0082 | 0.014 | 0.25 |
| dat.SOC1 | 0.83 | 0.83 | 0.84 | 0.28 | 4.8 | 0.0071 | 0.013 | 0.29 |
| dat.SOC4 | 0.81 | 0.81 | 0.82 | 0.26 | 4.3 | 0.0078 | 0.014 | 0.26 |
| dat.SOC7 | 0.81 | 0.81 | 0.82 | 0.26 | 4.2 | 0.0079 | 0.016 | 0.27 |
| dat.SOC12 | 0.81 | 0.81 | 0.82 | 0.26 | 4.2 | 0.0080 | 0.015 | 0.26 |

Item statistics

|  | n | raw r | std r | r cor | r drop | mean | sd |
| --- | --- | --- | --- | --- | --- | --- | --- |
| dat.SOC2 | 1235 | 0.42 | 0.43 | 0.36 | 0.31 | 3.5 | 2.1 |
| dat.SOC6 | 1235 | 0.60 | 0.59 | 0.55 | 0.50 | 4.2 | 2.2 |
| dat.SOC8 | 1235 | 0.73 | 0.72 | 0.72 | 0.65 | 3.5 | 2.3 |
| dat.SOC9 | 1235 | 0.71 | 0.70 | 0.70 | 0.63 | 3.2 | 2.3 |
| dat.SOC11 | 1235 | 0.56 | 0.56 | 0.50 | 0.45 | 3.6 | 2.2 |
| dat.SOC3 | 1235 | 0.46 | 0.47 | 0.41 | 0.34 | 3.8 | 2.1 |
| dat.SOC5 | 1235 | 0.60 | 0.60 | 0.55 | 0.50 | 4.3 | 2.4 |
| dat.SOC10 | 1235 | 0.58 | 0.59 | 0.53 | 0.48 | 3.6 | 2.0 |
| dat.SOC13 | 1235 | 0.63 | 0.63 | 0.59 | 0.54 | 3.9 | 2.2 |
| dat.SOC1 | 1235 | 0.34 | 0.34 | 0.24 | 0.21 | 4.5 | 2.3 |
| dat.SOC4 | 1235 | 0.54 | 0.54 | 0.49 | 0.44 | 4.8 | 2.1 |
| dat.SOC7 | 1235 | 0.55 | 0.56 | 0.51 | 0.46 | 5.1 | 2.0 |
| dat.SOC12 | 1235 | 0.58 | 0.58 | 0.53 | 0.48 | 4.1 | 2.2 |

Non missing response frequency for each item

|  | 1 | 2 | 3 | 4 | 5 | 6 | 7 | miss |
| --- | --- | --- | --- | --- | --- | --- | --- | --- |
| dat.SOC2 | 0.28 | 0.08 | 0.14 | 0.17 | 0.11 | 0.11 | 0.11 | 0 |
| dat.SOC6 | 0.21 | 0.06 | 0.13 | 0.15 | 0.09 | 0.10 | 0.26 | 0 |
| dat.SOC8 | 0.36 | 0.07 | 0.11 | 0.12 | 0.08 | 0.08 | 0.18 | 0 |
| dat.SOC9 | 0.42 | 0.08 | 0.11 | 0.09 | 0.06 | 0.09 | 0.15 | 0 |
| dat.SOC11 | 0.31 | 0.08 | 0.09 | 0.16 | 0.12 | 0.06 | 0.18 | 0 |
| dat.SOC3 | 0.22 | 0.08 | 0.16 | 0.17 | 0.11 | 0.11 | 0.15 | 0 |
| dat.SOC5 | 0.23 | 0.07 | 0.09 | 0.11 | 0.09 | 0.09 | 0.31 | 0 |
| dat.SOC10 | 0.24 | 0.08 | 0.15 | 0.18 | 0.11 | 0.13 | 0.10 | 0 |
| dat.SOC13 | 0.25 | 0.08 | 0.13 | 0.14 | 0.09 | 0.11 | 0.20 | 0 |
| dat.SOC1 | 0.19 | 0.04 | 0.08 | 0.16 | 0.11 | 0.10 | 0.32 | 0 |
| dat.SOC4 | 0.13 | 0.05 | 0.07 | 0.14 | 0.14 | 0.13 | 0.34 | 0 |
| dat.SOC7 | 0.10 | 0.02 | 0.07 | 0.18 | 0.15 | 0.13 | 0.35 | 0 |
| dat.SOC12 | 0.20 | 0.09 | 0.14 | 0.15 | 0.09 | 0.08 | 0.25 | 0 |

Split half reliabilities

Maximum split half reliability (lambda 4) = 0.86

Guttman lambda 6 = 0.83

Average split half reliability = 0.81

Guttman lambda 3 (alpha) = 0.82

Guttman lambda 2 = 0.83

Minimum split half reliability (beta) = 0.69

Average interitem r = 0.26 with median = 0.27

Guttman bounds

L1 = 0.76

L2 = 0.83

L3 (alpha) = 0.82

L4 (max) = 0.86

L5 = 0.81

L6 (smc) = 0.83

TenBerge bounds

mu0 = 0.82 mu1 = 0.83 mu2 = 0.83 mu3 = 0.83

alpha of first PC = 0.83

estimated greatest lower bound based upon communalities= 0.89

beta found by splitHalf = 0.69
