# Supplementary material for: Validation of the factor structure and psychometric characteristics of the Arabic adaptation of the sense of coherence SOC-13 scale: a confirmatory factor analysis
Source: BMC Psychol. 2022 May 3;10:115. doi: 10.1186/s40359-022-00826-4 (PMC9066761; doi:10.1186/s40359-022-00826-4)
Supplement: Supplementary file 2 — Additional file 2. Detailed account of the four-factor loading for the Arabic SOC-13 data. [file 40359_2022_826_MOESM2_ESM.docx]

**Validation of the Factor Structure and Psychometric Characteristics of the Arabic Adaptation of the Senses of Coherence SOC-13 Scale: a Confirmatory Factor Analysis**

# Authors:

Dr Fatimah Sayer Alharbi,

Assistant Professor in Mental Health Psychology, Nora University

Abdulaziz I Aljemaiah,

Senior Clinical Psychologist, Armed Forces Centre for Psychiatric Care, Taif, Saudi Arabia

Dr Mugtaba Osman,

Consultant Psychiatrist, Armed Forces Centre for Psychiatric Care, Taif, Saudi Arabia

Corresponding Author: Dr Mugtaba Osman, Email: [Mugtaba.osman@ucdconnect.ie](mailto:Mugtaba.osman@ucdconnect.ie), Address: Armed Forces Centre for Psychiatric Care, Prince Mansour Military Hospital, Al-Matar Street, Al-Faiysaliyah District, Taif, Saudi Arabia

Exploratory factor analysis


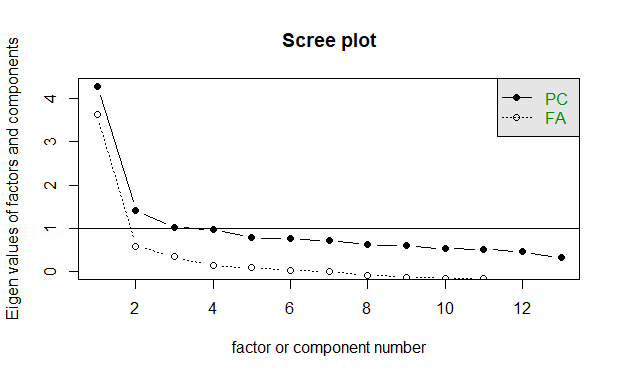


Scree plot gives a primary indication that a three-factor model should be sufficient.

Factor loading of the Three-Factor Model

Uniquenesses:

SOC1 SOC2 SOC3 SOC4 SOC5 SOC6 SOC7 SOC8 SOC9 SOC10 SOC11 SOC12 SOC13

0.945 0.528 0.499 0.543 0.628 0.674 0.666 0.389 0.280 0.694 0.764 0.699 0.622

Loadings:

Factor1 Factor2 Factor3

SOC1 0.164 0.137

SOC2 0.105 0.678

SOC3 0.134 0.101 0.687

SOC4 0.198 0.645

SOC5 0.234 0.535 0.178

SOC6 0.330 0.459

SOC7 0.151 0.525 0.188

SOC8 0.684 0.322 0.197

SOC9 0.806 0.229 0.135

SOC10 0.400 0.195 0.329

SOC11 0.363 0.300 0.119

SOC12 0.331 0.432

SOC13 0.505 0.341

Factor1 Factor2 Factor3

SS loadings 2.055 1.797 1.216

Proportion Var 0.158 0.138 0.094

Cumulative Var 0.158 0.296 0.390

Test of the hypothesis that 3 factors are sufficient.

The chi square statistic is 197.47 on 42 degrees of freedom.

The p-value is 5.24e-22

Factor loadings of the Four-Factor Model

Uniquenesses:

SOC1 SOC2 SOC3 SOC4 SOC5 SOC6 SOC7 SOC8 SOC9 SOC10 SOC11 SOC12 SOC13

0.944 0.552 0.473 0.450 0.637 0.635 0.659 0.455 0.005 0.699 0.695 0.670 0.560

Loadings:

Factor1 Factor2 Factor3 Factor4

SOC1 0.160 0.138

SOC2 0.106 0.659

SOC3 0.113 0.709

SOC4 0.204 0.698 0.135

SOC5 0.299 0.477 0.181 0.113

SOC6 0.487 0.332 0.104

SOC7 0.192 0.511 0.192

SOC8 0.444 0.297 0.227 0.457

SOC9 0.377 0.190 0.147 0.892

SOC10 0.321 0.144 0.342 0.246

SOC11 0.498 0.155 0.119 0.137

SOC12 0.465 0.298 0.140

SOC13 0.576 0.194 0.252

Factor1 Factor2 Factor3 Factor4

SS loadings 1.685 1.399 1.251 1.232

Proportion Var 0.130 0.108 0.096 0.095

Cumulative Var 0.130 0.237 0.333 0.428

Test of the hypothesis that 4 factors are sufficient.

The chi square statistic is 116.72 on 32 degrees of freedom.

The p-value is 1.43e-11

Factor loading of the truncated Three-Factor Model

Uniquenesses:

SOC4 SOC5 SOC6 SOC7 SOC8 SOC9 SOC10 SOC11 SOC12 SOC13

0.542 0.629 0.660 0.672 0.370 0.281 0.751 0.691 0.637 0.550

Loadings:

Factor1 Factor2 Factor3

SOC4 0.161 0.632 0.182

SOC5 0.228 0.522 0.216

SOC6 0.240 0.368 0.384

SOC7 0.159 0.524 0.167

SOC8 0.681 0.309 0.266

SOC9 0.768 0.202 0.298

SOC10 0.404 0.179 0.232

SOC11 0.245 0.171 0.469

SOC12 0.200 0.310 0.476

SOC13 0.373 0.199 0.520

Factor1 Factor2 Factor3

SS loadings 1.616 1.415 1.185

Proportion Var 0.162 0.141 0.119

Cumulative Var 0.162 0.303 0.422

Test of the hypothesis that 3 factors are sufficient.

The chi square statistic is 109.07 on 18 degrees of freedom.

The p-value is 4.68e-15
